# Investigating the Dietary Intake Using the CyFFQ Semi-Quantitative Food Frequency Questionnaire in Cypriot Huntington’s Disease Patients

**DOI:** 10.3390/nu15051136

**Published:** 2023-02-23

**Authors:** Christiana C. Christodoulou, Christiana A. Demetriou, Elena Philippou, Eleni Zamba Papanicolaou

**Affiliations:** 1 Neuroepidemiology Department, The Cyprus Institute of Neurology and Genetics, Nicosia 2371, Cyprus; 2 Department of Primary Care and Population Health, University of Nicosia Medical School, Nicosia 2371, Cyprus; 3 Department of Life Sciences, School of Life and Health Sciences, University of Nicosia, Nicosia 2417, Cyprus; 4 Department of Nutritional Sciences, King’s College London, London WC2R 2LS, UK

**Keywords:** Huntington’s Disease, food frequency questionnaire, CyFFQ, dietary assessment, Mediterranean Diet Adherence

## Abstract

Huntington’s disease (HD) is a rare progressive neurodegenerative disease characterised by autosomal dominant inheritance. The past decade saw a growing interest in the associations between the Mediterranean Diet (MD) and HD risk and outcomes. The aim of this case-control study was to assess the dietary intake and habits of Cypriot HD patients, comparing them to gender and age-matched controls, using the Cyprus Food Frequency Questionnaire (CyFFQ) and to assess adherence to the MD by disease outcomes. The method relied on the validated CyFFQ semi-quantitative questionnaire to assess energy, macro- and micronutrient intake over the past year in *n* = 36 cases and *n* = 37 controls. The MedDiet Score and the MEDAS score were used to assess adherence to the MD. Patients were grouped based on symptomatology such as movement and cognitive and behavioral impairment. The two-sample Wilcoxon rank-sum (Mann–Whitney) test was used to compare cases vs. controls. Statistically significant results were obtained for energy intake (kcal/day) (median (IQR): 4592 (3376) vs. 2488 (1917); *p* = 0.002) from cases and controls. Energy intake (kcal/day) (median (IQR): 3751 (1894) vs. 2488 (1917); *p* = 0.044) was also found to be significantly different between asymptomatic HD patients and controls. Symptomatic patients were also different from controls in terms of energy intake (kcal/day) (median (IQR): 5571 (2907) vs. 2488 (1917); *p* = 0.001); % energy monounsaturated fatty acids (median (IQR): 13.4 (5.2) vs. 15.5 (5.7); *p* = 0.0261) and several micronutrients. A significant difference between asymptomatic and symptomatic HD patients was seen in the MedDiet score (median (IQR): 31.1 (6.1) vs. 33.1 (8.1); *p* = 0.024) and a significant difference was observed between asymptomatic HD patient and controls (median (IQR): 5.5 (3.0) vs. 8.2 (2.0); *p* = 0.014) in the MEDAS score. This study confirmed previous findings that HD cases have a significantly higher energy intake than controls, revealing differences in macro and micronutrients and adherence to the MD by both patients and controls and by HD symptom severity. These findings are important as they are an effort to guide nutritional education within this population group and further understand diet–disease associations.

## 1. Introduction

Huntington’s Disease (HD) is a rare and progressive neurodegenerative disease characterised by autosomal dominant inheritance, affecting the medium spiny neurons of the basal ganglia [1,2]. The mean age of onset is approximately 40 years of age [1] and clinical features include movement (incoordination), cognitive (lapse in short-term memory) and behavioural (depression) impairments [3]. HD is caused by a mutation of the Huntington (*HTT*) gene, which is located on chromosome 4 of exon 1 and, more specifically, a CAG trinucleotide repeat expansion at the N-terminus of the *HTT* gene [4]. The number of CAG repeats is the main predictor for the age of onset, disease severity and occurrence of HD. While the CAG trinucleotide is repeated between 10–35 times in healthy individuals, HD individuals can have from 36–120 CAG repeats. Individuals that have between 36–39 CAG repeats may or may not develop signs and symptoms of the disease, meaning that there is a reduced penetrance [5]. Individuals, however, with 40 or more repeats will always develop signs and symptoms of HD [5]. The trinucleotide repeat varies in length among individuals and among generations [5]. Despite the number of CAG repeats being the major determinant of the age of onset, there is still variation in the age of disease onset among individuals with the same number of repeats. This is a finding that remains unexplained [5]. Furthermore, although HD is a monogenic disease, its molecular manifestations seem highly complex and involve multiple cellular processes [5].

In Cyprus, in 2015, the prevalence and incidence of symptomatic HD were 4.64 per 100,000 population (95% CI: 3.30–6.34) and 0.12 per 100,000 population (95% CI: 0.00–0.66), respectively [6]. The frequency of individuals with a pathogenic triplet expansion in the population at the end of 2014 was estimated to be 14.1 per 100,000 population [6]. In other words, 1 in 7097 individuals was expected to have one allele with a pathogenic CAG repeat range, which translates to roughly 120 heterozygotes in the population in the areas controlled by the Republic of Cyprus. These rates are comparable to other European Countries [6]. 

A nutritional assessment that considers the disease stage and feeding difficulties in HD patients is important due to a high prevalence of malnutrition, as evidenced by lower-than-average body weight in many of these patients [7,8]. Due to the variability in energy requirements and rapid weight loss, early assessment and regular reviewing of nutritional care plans are fundamental [8]. This calls for frequent monitoring of the patients’ body weight and adjustment of their energy intake to reach the ideal or target body weight. Furthermore, many HD patients have increased energy requirements either due to motor impairment or having a hypermetabolic state, defined as an elevated resting energy expenditure. Therefore, it is essential to provide adequate macro and micronutrients [8]. Nutritional education should be central to disease management both for the patients themselves and their families since this can guide them in choosing a healthier diet, address nutritional issues of concern depending on the disease stage and reduce the risk of malnutrition [9].

Previous studies have investigated dietary intake and the effect of adhering to the Mediterranean Diet (MD) adherence in delaying disease progression, improving the Unified Huntington’s Disease Rating Scale (UHDRS) score as well as improving motor function and cognition in HD patients, as recently published in our systematic review (SLR) [10]. In this SLR, a total of 18 studies, including randomized controlled trials and non-randomized intervention trials, case-control studies and cohort studies, were carried out. The studies investigated (i) dietary intake and patterns, (ii) MD adherence, (iii) nutritional supplementation, and (iv) caloric intake in individuals with HD. The findings suggested an improvement in the motor and cognitive scores and a better quality of life in people with HD with higher MD adherence [10]. Furthermore, a high energy intake was repeatedly observed in people with HD, likely due to the higher energy consumption [10]. Moreover, certain food groups, such as milk and dairy products and caffeine consumption, greater than 190 mg/day were found to be associated with an earlier age of disease onset [10], although these findings need further investigation.

There is limited research on energy and macronutrient intake in HD patients, with only two studies being identified. Marder et al., 2009 [11] conducted a case-control study that investigated energy, macronutrient intake and body mass index (BMI) in 217 HD carriers with expanded CAG ≥ 37 and 435 non-expanded CAG < 37 HD carriers and controls [10,11]. Individuals with expanded CAG ≥ 37 had significantly higher UHDRS motor scores compared to non-expanded CAG < 37 HD. Energy intake was strongly associated with CAG repeat length and with the estimated 5-year probability for HD onset in the expanded CAG ≥ 37 group. Increased caloric intake may be necessary to maintain BMI in asymptomatic HD individuals with CAG ≥ 37. This may be related to increased energy expenditure as a result of subtle motor impairment or a hypermetabolic state [10,11]. Furthermore, carbohydrate intake was also significantly higher in the expanded CAG ≥ 37 group compared to the non-expanded CAG < 37 HD carriers and control group.

A case-control study including 32 individuals with an abnormal CAG repeat length (>36) (15 asymptomatic and 17 HD patients) and 21 controls by Mochel et al., 2007 [10] performed a multi-parametric study to investigate body weight and mechanisms of body weight loss [10]. A semi-quantitative questionnaire inquiring about regular food and beverage consumption was used to observe energy intake for 24 h in HD patients [10]. Body weight change was determined by subtracting current weight from the recorded weight in the medical records of each participant 5 years prior to study inclusion. HD patients were found to have significant weight loss compared to controls. Furthermore, men with HD had lower BMI compared to controls, while total energy intake was inversely associated with weight and lean body mass, indicating that people with HD exhibit an early hypermetabolic state due to increased energy expenditure. Weight loss was also observed in asymptomatic carriers, even though they had higher energy intake compared to controls [10,12].

Over the past decade, there is growing scientific evidence indicating the beneficial effects of the MD in a number of diseases, including neurodegenerative disease (ND), cardiovascular, type II diabetes mellitus and obesity [13,14]. The MD is a dietary pattern that originated from the regions of the Mediterranean basin e.g., Italy, Spain, Portugal, Greece and Cyprus. It is characterized by: a high intake of whole grains, legumes, fruit, vegetables, nuts and seeds, fresh herbs and spices and the use of extra virgin olive oil as the main source of fat; a moderate consumption of dairy products, poultry, fish, seafood and eggs; a limited consumption of red meat and desserts; and moderate alcohol intake, mainly red wine with meals [15]. Plant-based foods, such as whole grains, legumes, fruits, vegetables and olive oil, are rich sources of phytochemicals, carotenoids, flavonoids, vitamins and minerals with anti-oxidative, neuro-protective and anti-inflammatory properties preventing reactive oxidative stress (ROS) and lipid and protein damage [15]. Interestingly, in recent years, the effects of the MD on the prevention of NDs such as Alzheimer’s and Parkinson’s disease have been studied [14]. Long-term consumption of the MD not only reduces ROS and neuroinflammation in ND, but leads to an increase of longevity via the maintenance of telomere length and prevention of brain atrophy [15]. As in other NDs, the MD may also be protective in HD patients. Indeed, some evidence suggests that higher adherence to the MD is associated with a better-quality diet and patient outcomes. A study by Rivadeneyra et al., 2016 [16] observed that HD patients with high or moderate MD adherence had increased micronutrient intake compared to those with low MD adherence [10,16]. Moderate/high MD adherence was characterized by a higher intake of monounsaturated fatty acids (MUFA), saturated fatty acids (SFA) and polyunsaturated (PUFA) + MUFA/SFA, which was associated with a slight improvement of Total Functional Capacity (TFC) and UHDRS cognitive scores compared to low MD adherence. Furthermore, moderate to high MD adherence was associated with a slight improvement in UHDRS motor and cognitive scores. However, HD severity was similar between subjects with low vs. moderate/high MD adherence [16].

Even though adherence to the MD has been shown to be beneficial in HD patients, there is a lack of research investigating dietary intake and MD adherence using validated food frequency questionnaires (FFQs), which assess macro- and micronutrient intake in HD patients with different symptomatology and disease stage. The present case-control study aimed to perform this by further categorizing patients by HD stages, namely: asymptomatic, symptomatic and symptomatic advanced. The inclusion of three HD stages is not easily observed among research studies in HD either, due to the small sample size or lack of participants in each disease stage. Including patients from all disease stages may lead to a better understanding of the association between their dietary intake, including MD adherence, and HD symptoms. Furthermore, to the best of our knowledge, there are no studies investigating dietary intake and MD adherence in Cypriot HD patients in comparison to matched controls. 

Additionally, our study used the Cyprus FFQ (CyFFQ), a semi-quantitative validated FFQ, for assessing the dietary intake of the Cypriot population, which provides a detailed assessment of culture-specific intake and Western foods [17]. The present investigation thus aimed to investigate the dietary intake and MD adherence scores of Cypriot HD patients who are either (i) asymptomatic, (ii) symptomatic and (iii) symptomatic advanced versus gender/age-matched controls using the CyFFQ to identify any associations between MD adherence and disease stage. It was hypothesized that high adherence to the MD would be associated with delayed disease symptoms and a better quality of life due to higher consumption of neuroprotective and anti-inflammatory foods. 

## 2. Materials and Methods

**Figure 1:**
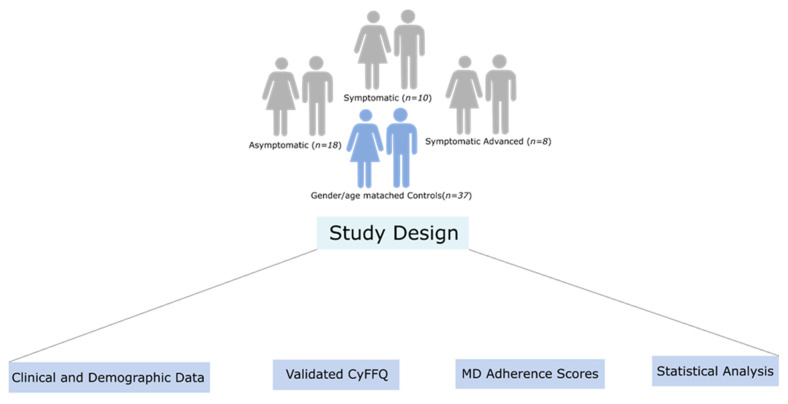
Workflow of dietary assessment in Cypriot HD patients versus gender/age-matched controls.

The workflow implemented in our study is illustrated in Figure 1 and details regarding each step of the study workflow are described below. 

### 2.1. Study Design

All HD patients being cared for by the Cyprus Institute of Neurology and Genetics (CING), a referral center for HD in Cyprus, were invited to participate in the study by their neurologist (E.Z.P). Participant recruitment occurred between November 2017 to March 2019. The age of recruitment for HD patients was between 18–75 years old. In addition, healthy gender/age-matched controls were recruited from HD families (members without the pathological CAG trinucleotide expansion), patients’ carers and CING staff members. Each HD patient was matched with a control based on age and gender. All participants were from the area under the control of the Republic of Cyprus.

Demographic and lifestyle information, including diet and medical history, was obtained through an interviewer-administered questionnaire. 

The information collected is shown below:

- Demographics: sex, date of birth, birthplace and current city of residence, parents’ birthplace, family status (single, married, divorced or widowed) and occupation;
- Medical and family history of HD and their family as well as presence and age of HD symptoms family members diagnosed with HD and other chronic illnesses for patients and controls;
- Age of onset and symptoms, number of CAG repeat and current medical treatment;
- Anthropometrics: weight and height at least a year prior to HD diagnosis;
- Lifestyle, such as smoking, current smoking, physical exercise and hobbies;
- Medical history, CAG repeat counts, treatments and other co-morbidities were also obtained from the patients’ medical records, or via self-reporting for controls.

The study was reviewed and ethically approved by the Cyprus National Bioethics Committee (EEBK/EP/2017/29) and conducted in accordance with the Declaration of Helsinki. Written, informed consent was obtained from all participants prior to the study participation. 

### 2.2. Huntington’s Disease Assessment

The Unified Huntington’s Disease Rating Scale (UHDRS) was used to assess four domains of clinical performance and capacity in HD, these being motor and cognitive function, behavioral abnormalities and functional capacity [18]. Asymptomatic and symptomatic HD patients were categorized based on their UHDRS scores. Patients were categorized as symptomatic based on their scores; higher scores indicate the inability to perform motor tasks and behavioral impairment while lower scores are an indication of cognitive impairment and a decrease in functional capacity [18]. 

### 2.3. Food Frequency Questionnaire

The validated semi-quantitative CyFFQ was developed by two experienced dietitians as described by Philippou et al., 2022 [17]. It consisted of 171 food items that reflected the dietary intake of commonly consumed foods within the Cypriot population over the previous year. The average energy, macro-and micronutrient intake is calculated as previously explained in detail [17]. 

In brief, the CyFFQ was interview-administered and the participant had to respond for each food item with reference to frequency of consumption during the previous year (nine possible responses ranging from never to every day) and the amount consumed, using either the Greek translation of the Block Portion Size Picture© (used with permission after the purchase of copyrights from NutritionQuest©) (https://www.nutritionquest.com/) (accessed on 1 September 2017), tablespoons/teaspoons or the number of items, depending on the food item. In addition to the items included in the CyFFQ, participants had the opportunity to report foods and beverages usually consumed, using open-ended questions. It is of note that items other than the ones in the main FFQ were rarely reported. The FFQ administration and completion varied from approximately 1–2 h, depending on the study participant. The analysis of the CyFFQ was performed using the Dietplan7 software (https://www.foresoft.co.uk/) (accessed on 5 May 2018) to which traditional food items were added, as described elsewhere [17].

### 2.4. Presentation of FFQ Data

The macronutrients were expressed as percentages of energy intake and grams, while the micronutrients were expressed as g or mg/1000 kcal. 

### 2.5. Comparison of Intake with Dietary Reference Intakes

The participants’ intake grouped by life stage and gender was compared against the Dietary Reference Intake (DRIs) [19].

### 2.6. MD Adherence Scores

MD adherence was evaluated based on the CyFFQ dietary intake reports of individuals using the following scores: the MedDiet score by Panagiotakos et al., 2007 [20] and the Mediterranean Diet Adherence Screener (MEDAS) score proposed by Martínez-González et al., 2004 [21], which was validated for the Cypriot population [17]. The MedDiet score includes 11 food groups and was developed to estimate the adherence to MD and its association with cardiovascular disease risk and biomarkers [20], while the MEDAS consists of a 14-food item questionnaire.

For the MedDiet score, food items that are regularly consumed within the MD dietary pattern, such as non-refined cereals, fruits, vegetables, legumes, olive oil, fish and potatoes, were assigned a score ranging from 1 to 5, indicating rare or no consumption to daily consumption [20]. The reverse scores were assigned for the consumption of foods that deviated from the MD dietary pattern, such as meat and meat products. A score of 5 was assigned when individuals reported no consumption of the specified food group and 0 for daily consumption [20]. In the case of alcohol consumption, the following scoring method was assigned: a score of 5 for <300 mL of alcohol/day, a score of 0 for >700 mL per day or no consumption, scores of 4 to 1 for consumption of 600–700 mL/day, 500–600 mL/day, 400–500 mL/day and 300–400 mL/day respectively. In total, the lowest possible score for the MedDiet score is 0 and the maximum is 55, with the highest score indicating a higher adherence to the MD diet. 

For the MEDAS score [21], a score of 1 is given for the consumption of a beneficial food item above a certain frequency and a score of 0 is given if the consumption of a beneficial food item is below the required intake. The total MEDAS score ranges from 0 to 14, and a higher score indicates better MD adherence [21]. (The criteria and scoring for each approach are presented in Tables S4 and S5 in the Supplementary Material). 

The MD adherence scores were derived from the FFQs by grouping foods based on the main categories assessed by each MD adherence score. As an example, the legume group included lima beans, chickpeas, lentils, green peas and black-eyed beans. Grouping was performed by trained researchers (C.C.C and C.A.D). 

### 2.7. Statistical Analysis

All statistical analyses were performed using the STATA statistical software, version SE16 (StatCorp. 2007. College Station, TX, USA) and R studio, using R statistical packages and scripts, version 3.6.1 (R Core Team, R Foundation for Statistical Computing, Vienna, Austria). Descriptive statistical analyses of mean and standard deviation and median and interquartile range (minimum and maximum) were applied to the energy-adjusted values obtained from the FFQ for cases and controls. Additionally, a statistical analysis comparing the energy-adjusted values for controls versus asymptomatic HD patients and for controls versus symptomatic HD patients was carried out using the two-sample Wilcoxon rank-sum (Mann–Whitney) test. 

Statistical analysis also compared macronutrient intakes in cases and controls against the recommended gender and age-specific macronutrient intakes. The Fisher’s exact test was used to calculate a *p*-value comparing the percentages of each group that were within or outside the recommendation range. 

Comparisons between the MedDiet and the MEDAS scores, asymptomatic patients and controls, symptomatic patients and controls and asymptomatic and symptomatic HD patients were performed using the two–sample Wilcoxon rank-sum (Mann–Whitney). For all analyses, a *p*-value of <0.05 was considered statistically significant. 

## 3. Results

### 3.1. Demographic and Anthropometric Characteristics of Participants

This case-control study consisted of 36 HD patients, of which *n* = 18 were asymptomatic, *n* = 10 were symptomatic and *n* = 8 were symptomatic advanced-stage HD patients, as well as their gender/age-matched controls (*n* = 37). 

The demographic characteristics of participants are shown in the Supplementary Material as Supplementary Tables S1 and S2. Overall, the three study groups were similar in terms of their demographics, with a few exceptions. There was a statistically significant difference between the educational level of the asymptomatic HD patients versus controls, with a higher percentage of asymptomatic HD patients having completed higher levels of education [primary school (1% asymptomatic vs. 2% controls), lower secondary school (5% asymptomatic vs. 5% controls), high school (4% asymptomatic vs. 6% controls) and higher education (25% asymptomatic vs. 5% controls) (*p* = 0.014)]. 

A statistically significant difference in age was identified between asymptomatic HD patients versus controls (*p* = 0.026), symptomatic HD patients versus controls (*p* = 0.041) and asymptomatic HD patients versus symptomatic HD patients (*p* = <0.00001) Supplementary Tables S1–S3). There was a statistically significant difference between weight in controls versus symptomatic HD patients (*p* = 0.030) (Supplementary Table S2). However, no significant difference was identified for controls versus asymptomatic HD patients and between the asymptomatic and symptomatic HD patients. Furthermore, the body mass index (BMI) did not differ significantly between all three groups (Supplementary Tables S1–S3). 

A statistically significant difference was also identified for marital status between symptomatic HD patients versus controls, with more controls being married (9% symptomatic vs. 26% controls), single (4% symptomatic vs. 8% controls), divorced (3% symptomatic vs. 0% controls) and widowed (1% symptomatic vs. 2% controls) (*p* = 0.035)] (Supplementary Table S2). 

### 3.2. Energy Intake, EA, Macronutrients and Micronutrients

As seen in Table 1, energy intake (kcal/day) was higher in cases vs. controls (median (IQR): 4592 (3376) vs. 2488 (1917) kcal/day; *p* = 0.0002). Additionally, cases consumed a lower percentage of energy from non-starch polysaccharides (NSPs) compared to controls (median (IQR): 5.5 (3.2) vs. 8.1 (4.1)% energy NSPs; *p* = 0.034). 

A comparison of the EA macronutrients and micronutrients between asymptomatic HD (*n* = 18) and controls (*n* = 37) was also performed as shown in Table 2. Cases had a higher energy intake (median (IQR): 3751 (1894) kcal/day) compared to controls (2488 (1917) kcal/day; *p* = 0.028). Additionally, the following macronutrients and minerals were significantly different between asymptomatic HD patients vs. controls, respectively: % energy of polyunsaturated fatty acids (PUFAs) (median (IQR): 7.5 (2.6) vs. 6.4 (1.7)% energy; *p* = 0.032); cholesterol (median (IQR): 135.8 (55.4) vs. 106.8 (58.8) mg/1000 kcal; *p* = 0.023); sodium (median (IQR: 1028.6 (290.2) vs. 915.9 (344.0) mg/1000 kcal; *p* = 0.044); selenium (median (IQR: 19.7 (6.8) vs. 16.9 (6.4) ug/1000 kcal; *p* = 0.023); and tryptophan (median and (IQR): 6.8 (2.1) vs. 5.7 (1.7) mg/1000 kcal; *p* = 0.007). 

A comparison of the macronutrient and micronutrient intake between controls (*n* = 37) and symptomatic HD (*n* = 18) was also performed as shown in Table 3. The symptomatic and advanced symptomatic patient groups were grouped together for this analysis. Energy (kcal/day) was found to be statistically significantly different between symptomatic HD patients and controls (median and (IQR): 5517 (2907) vs. 2488 (1917) kcal/day; *p* = 0.001). The following dietary macronutrients and micronutrients were significantly different in symptomatic HD patients versus controls, respectively: % energy monounsaturated fatty acids (MUFA) (median and (IQR): 13.4 (5.2) vs. 15.5 (5.7) % energy; *p* = 0.026); % energy NSPs (median and (IQR): 1.09 (0.57) vs. 1.62 (0.81)% energy; *p* = 0.043); potassium (median and (IQR): 1155.7 (185.3) vs. 1377.8 (292.0) mg/1000 kcal; *p* = 0.017); calcium (median and (IQR): 348.2 (99.3) vs. 402.6 (159.4) mg/1000 kcal; *p* = 0.024); magnesium (median and (IQR): 115.8 (38.1) vs. 141.7 (33.7) mg/1000 kcal; *p* = 0.023); phosphorus (median and (IQR): 503.8 (84.2) vs. 619.8 (158.4) mg/1000 kcal; *p* = 0.002); and biotin (median and (IQR): 8.1 (2.2) vs. 9.4 (3.5) ug/1000 kcal; *p* = 0.021). 

A comparison of adherence to macro and micronutrient intake recommendations between cases (*n* = 36) versus controls (*n* = 37) was also performed, as shown in Table 4. Cases showed higher adherence to recommendations for magnesium, vitamin E, vitamin B6, folate and pantothenic acid. Controls demonstrated a higher percentage of adherence to the recommendations of macro and micronutrients compared to symptomatic HD patients regarding energy, cholesterol, sodium and chloride. With regards to a higher adherence in cases vs. controls, in particular, the percentage (lower 95% CI-Upper 95% CI) of cases that fell within the recommended range for magnesium was: 80.6 (63.8–90.7) vs. controls: 56.8 (40.1–72.0)%; *p* = 0.043); % within recommendation for vitamin E (cases: 61.1 (44.0–75.9) vs. controls: 21.6 (10.9–38.3)%; *p* = 0.010); % within recommendation for vitamin B6 cases: 94.4 (79.5–98.7) vs. controls: 75.7 (58.9–87.1)%; *p* = 0.046); % within recommendation for folate cases: 33.3 (19.6–50.6) vs. controls: 10.8 (4.0–26.2)%; *p* = 0.025); and % within recommendation for pantothenic acid cases: 86.1 (70.0–94.3) vs. controls: 51.4 (35.1–67.3)%; *p* = 0.002). All were significantly higher for the case compared to the control group. In contrast, adherence to other dietary recommendations was higher in controls compared to cases. In particular, % within recommendation for energy intake (kcal/day) of cases vs. controls: 8.3 (2.6–23.6) vs. 29.7 (16.9–46.7)%; *p* = 0.035); % within recommendation for cholesterol intake cases: 8.3 (2.6–23.6) vs. controls: 51.4 (35.1–67.3)%; *p* = <0.0001); % within recommendation for sodium cases: 8.3 (2.6–23.6) vs. controls: 45.9 (30.3–62.4)%; *p* = <0.0001); and % within recommendation for chloride intake cases: 13.9 (5.7–30.0) vs. controls: 51.4 (35.1–67.3)%; *p* = 0.001). 

### 3.3. MD Adherence Scores Using the MedDiet Score and the MEDAS Score

As seen in Table 5, no significant difference in MD adherence assessed using the MedDiet score was observed when comparing asymptomatic individuals and controls (median and (interquartile range (IQR)): 31.1 (6.1) vs. 31.1 (8.1); *p* = 0.363), nor between symptomatic individuals and controls (median and (IQR): 33.1 (6.1) vs. 31.1 (8.1); *p* = 0.061). However, a significant difference was identified for MD adherence for asymptomatic individuals and symptomatic individuals (median and (IQR): 31.1 (6.1) vs. 33.1 (8.1); *p* = 0.024). Symptomatic patients had, on average, higher adherence to the MD than asymptomatic patients (Table 5). 

As seen in Table 6, the assessment of MD adherence using the MEDAS score revealed a significant difference in MD adherence between asymptomatic individuals and controls (median and (IQR): 5.5 (3.0) vs. 8.2 (2.0); *p* = 0.014), with controls adhering more to the MD than asymptomatic individuals. However, no significant difference was observed between symptomatic individuals and controls (median and (IQR): 6.2 (1.0) vs. 8.2 (2.0); *p* = 0.066) or between asymptomatic individuals and symptomatic individuals (median and (IQR): 5.5 (3.0) vs. 6.2 (1.0); *p* = 0.216).

## 4. Discussion

The present study investigated energy, macro and micronutrient intake and MD adherence in Cypriot HD patients who were either asymptomatic, symptomatic or symptomatic advanced versus age and gender-matched controls using the validated semi-quantitative CyFFQ and MD adherence scores. 

In brief, HD cases were found to have significantly higher energy intake compared to controls. This was observed for asymptomatic vs. controls and symptomatic vs. controls. The intake of asymptomatic HD was significantly higher regarding intakes of PUFA, cholesterol, sodium, selenium and tryptophan compared to controls, whereas intakes of MUFA, NSPs, potassium, calcium, magnesium, phosphorus and biotin were significantly higher in symptomatic HD versus controls. Symptomatic patients had higher MD adherence compared to asymptomatic patients based on the MedDiet score, while the MEDAS score controls demonstrated higher MD adherence compared to asymptomatic individuals. The BMI status was also investigated for controls versus asymptomatic HD patients, controls versus symptomatic HD patients and asymptomatic HD patients versus symptomatic HD patients. However, no statistically significant results were identified. Thus, although HD patients have more caloric intake, this does not translate to a higher BMI. In fact, HD patients have a significantly lower weight than controls.

Previous studies have investigated different dietary supplementations, such as ethyl-EPA [22,23], L-acetyl-carnitne (LACC) [24], uric acid [25], nutritional and oral supplementation of vitamins and minerals [26,27], to assess whether these improve the motor function of individuals with HD. 

The studies by Puri et al. [22,23] investigated the effect of ethyl-EPA in terms of motor improvement between the intent to treat (ITT) and protocol violations (PP) in cohort patient groups. However, no motor improvement was observed in the ITT-treated-group. However, in the protocol violations (PP) cohort, the ethyl-EPA group showed motor improvement in comparison to the placebo group [22,23]. Another study with ethyl-EPA supplementation [22,23] showed that ethyl-EPA was effective in significantly reducing global cerebral atrophy during the first month of treatment. More precisely, a decrease of the caudate and thalamus regions was observed in the ethyl-EPA-treated group. Therefore, ethyl-EPA shows some beneficial effects on reducing brain atrophy and improving motor function [22,23].

The relationship between uric acid (UA) and the progression of HD was investigated by looking at the functional decline in people with HD [25]. An association was observed between baseline UA and total functional capacity (TFC) over a 30-month period, from the lowest to highest quintile. More precisely, increasing UA levels were associated with less decline in the total motor scores from the lowest to the highest quintile. This suggests an association between the baseline UA concentration and slower progression of HD [25]. 

A study by Auinger et al. [26,27] investigated the use of oral nutritional supplements in HD patients [26,27]. The dietary assessment of macronutrients and energy intake was assessed in HD patients along with their UHDRS scores to monitor improvement in their motor, cognitive or behavioral domains. No change was observed in HD patients’ UHDRS scores from day 0 to day 90, indicating no association between diet and UHDRS scores [26,27]. An additional study [26,27] investigated oral supplementation of vitamins and minerals. The study found that a higher intake of water-soluble vitamins and minerals was more common in advanced HD patients. However, no significant benefit was observed with supplementation intake in terms of motor, cognitive or functional states of patients [26,27]. However, there are studies that investigated nutrient supplementation in HD. Some nutrients have demonstrated the ability to improve motor function or slow disease progression. Further research is required to better understand the effect of these vitamins and minerals on HD and their effect on improving HD disease symptoms and progression. 

The higher energy intake in HD patients compared to controls was not surprising and increased intake was observed as follows: symptomatic → asymptomatic → controls. Our results agree with previous studies that have identified that HD patients have an increased energy intake [11]. A previous study investigated the energy intake, BMI and macronutrient intake between 435 HD patients with <37 CAG repeats and 217 HD patients with ≥37 CAG repeats [11]. HD patients with ≥37 CAG repeats had higher energy intake compared to <37 CAG repeat HD patients. Furthermore, the ≥37 CAG HD group had a decreased BMI compared to the <37 CAG repeat group. Higher energy intake was related to both the increased CAG repeat length and the 5-year probability of HD onset. Additionally, the study did not find any differences in macronutrient intake following adjustment for confounders in the ≥37 CAG group compared to the <37 CAG group [11].

Another study [27] investigated the relationship between nutritional status and HD severity in 224 Spanish HD patients versus controls. This study revealed that the energy intake of 48 HD patients was below the recommended dietary allowance, while, in 150 HD patients, energy intake was higher than the recommended dietary intake [27]. Our study’s findings thus agree with previous studies arguing that HD patients have a higher energy intake compared to controls. The hypermetabolic state seen in HD patients is not explained by common pathophysiological mechanisms such as inflammation and altered endocrine function, although both mechanisms have been implicated in HD and are likely part of the pathological process induced by mutated huntingtin (mHTT) [12]. As a result, weight loss starts during the early stage of the disease and it is evident in asymptomatic patients despite high caloric intake.

A study by Mochel et al. [12] investigated early energy alternations in 32 asymptomatic and HD patients compared to 21 controls, revealing possible mechanisms which might explain the hypermetabolic state and weight loss observed in HD patients [12]. Nuclear magnetic resonance (NMR) conducted on the participants’ plasma identified low concentrations of the branched chain amino acids (BCAA), namely valine, leucine and isoleucine in HD patients compared to controls. BCAA levels were correlated with weight loss, disease progression and an abnormal CAG repeat expansion [12]. Therefore, the early weight loss seen in HD is associated with a systemic metabolic defect. BCAA concentrations may be indicative of disease onset and progression [12]. Further research can provide insight into the exact mechanisms contributing to weight loss observed in HD. 

In exploring energy dysfunction, brain, biochemical and cellular mechanisms have been identified. Brain energy metabolism revealed a decrease in glucose consumption in the basal ganglia and increases in lactate concentration in the basal ganglia and occipital cortex of HD patients [28]. Furthermore, ATP depletion was also seen in HD brain tissues. Various mechanisms have been proposed to explain the energy deficit in HD brains. These include impaired oxidative phosphorylation, oxidative distress, impaired mitochondrial calcium handling and a decrease in glycolysis, among others [28]. The biochemical evidence relating to energy deficit involves dysfunction in the respiratory chain complex II succinate dehydrogenase (SDH) [28]. Post-mortem studies of symptomatic HD patients showed a dysfunction in SDH (complex II) and Coenzyme Q-cytochrome c reductase (complex III) [28]. In a yeast model, it was observed that mHTT suppress mitochondrial respiration by suppressing succinate dehydrogenase and Coenzyme Q-cytochrome c reductase [28]. A possible cellular mechanism involved in energy deficit may be that mHTT impairs mitochondrial motility in mammalian neurons through a toxic gain of function from the CAG repeat expansion and loss of function of wild-type HTT [28]. Another possible explanation for energy dysfunction in HD is the downregulation of the peroxisome proliferator-activated receptor gamma coactivator (PGC-1a) in the striatum, which was shown to affect mitochondrial energy metabolism by impairing oxidative phosphorylation [28]. 

A few studies were conducted to investigate the nutritional status and severity in the Spanish HD population versus controls [27]. The study by Cubo et al. [27] identified possible dietary insufficiency in the intake of carbohydrates, PUFA, MUFA, fibre, Vitamin A, Vitamin E, pantothenic acid, biotin, folic acid, Vitamin D, iodine, potassium, copper and manganese, with the aforementioned macronutrients and micronutrients being below the recommended daily allowance [27]. Furthermore, symptomatic advanced HD patients demonstrated a higher intake of water-soluble vitamins such as Vitamin C, Vitamin B_2_, Vitamin B_6_, biotin and Vitamin B_5._ Our results found that MUFA, fibre, calcium, magnesium and biotin were significantly higher in controls compared to patients, suggesting a lower consumption of whole grains, legumes, eggs, meat, fruits and dark leafy vegetables by patients. Furthermore, PUFA intake was higher in asymptomatic HD patients and, since PUFA sources are usually rich in antioxidants, they may be providing neuronal protection by eliminating ROS, thus possibly delaying symptom onset in these patients. In contrast, intake of magnesium, zinc, selenium, Vitamin E, Vitamin B_6_, folate, biotin and Vitamin B_2_ was significantly higher in cases compared to controls. 

With regards to MD adherence, our study used two MD adherence scores, namely MedDiet and the MEDAS scores, to assess adherence. Using the MedDiet score, symptomatic HD patients demonstrated higher MD adherence compared to asymptomatic HD patients. The difference in MD adherence between the two HD stages may be the result of asymptomatic patients having less concern about their diet or using emotional eating to handle their diagnosis, as revealed by some patients and their families (unpublished data), while symptomatic patients may be considering healthy eating as a way to reduce symptomatology. A higher MD adherence was demonstrated in symptomatic HD patients compared to asymptomatic HD patients. A possible explanation is that asymptomatic HD patients consume more energy-dense foods, which may not be in accordance with the components of the MD. Indeed, since HD patients need to aim for a higher BMI, energy-dense foods such as full-fat dairy products, cream, red meat, fried foods and desserts may be preferred [24]. 

A previous study by Rivadeneyra et al. [16] investigated the factors associated with MD adherence in HD using the Trichopoulou score [16]. It revealed that HD patients with moderate to high MD adherence compared to those with low MD adherence had a higher intake of cereals, alcohol, fish, MUFA/SFA and dairy products [16]. Furthermore, moderate MD adherence was statistically significantly associated with old age, a decrease in comorbidities, UHDRS motor scores and lower abdominal obesity. In participants with high MD adherence, there was a decrease of UHDRS motor scores and psychiatric comorbidities and an improvement in the quality of life compared to those with lower MD adherence [16]. Our study results agree with the study by Rivadeneyra et al. [16] that the symptomatic HD patients have a higher MD adherence score compared to HD gene carriers and asymptomatic HD patients, although a different MD adherence score was used to assess this. 

With regard to adherence to the MD diet and dietary quality, a previous study investigated the dietary intake of HD patients based on their MD adherence [16]. Numerous vitamins and minerals, such as thiamin, biotin, folic acid, Vitamins A, C, D and E, phosphorus and potassium were studied in 98 HD patients who had low or moderate/high adherence to the MD. It revealed that biotin, folic acid, Vitamin C, Vitamin E, copper and selenium were significantly higher in the HD group with high/moderate compared to low MD adherence [16]. The results of the present study agree that the intake of some vitamins and minerals such as magnesium, zinc, Vitamin E and biotin were found to be significantly higher in HD patients compared to controls, suggesting that HD patients may be consuming foods higher in these micronutrients.

Although HD patients have higher energy intake, they often have lower-than-average body weight, struggle with malnutrition and may have an imbalance in food intake. Despite having higher energy intake, they may not meet energy requirements, since they often have lower- quality diet [29]. Insufficient energy, macro- and/or micronutrient intake leads to vitamin and mineral deficiencies such as anaemia and associated fatigue [29]. The HD patients’ inability to meet energy and nutrient requirements may be on account of (i) their not preferring certain foods or food groups such as fruits and vegetables, which are known to have antioxidant properties and are high in vitamins and minerals, (ii) having reduced or high appetite and (iii) chronic obsessive-compulsive tendencies towards certain foods or food groups for a long period of time [7,29]. The present study has a number of strengths and limitations. With regards to strengths, to the best of our knowledge, this is the first study in Cypriot HD patients that comprehensively assessed the dietary intake of this population group, both in terms of energy intake and macro and micronutrients, relying on the validated CyFFQ and adherence to the MD using two validated tools. The study included a high percentage of known HD patients in Cyprus and a matched control group. Generally, all the FFQs were completed except for one, which was partly completed. The study is limited because the sample size is small, due to the rarity of HD. Due to the cross-sectional nature of the study, nutritional changes could not be assessed in parallel with body composition, missing demographic and lifestyle data, since questionnaire completion was time-consuming and sometimes tiresome for some patients and their next of kin. Although completion of the CyFFQ typically takes 1 h, as mentioned by Philippou et al. [17] the next of kin of symptomatic HD patients sometimes took 2 h to complete it as they delved into more detail regarding food consumption, or they would be side-tracked and discuss other issues. This was not the case for asymptomatic and control participants. It is also of note that controls had a higher energy intake than expected, which might have deviated from some of the comparisons. A number of reasons may explain this, such as: (i) consumption of energy-dense meals and snacks contributing to higher energy intake; (ii) various snacking behaviors (eating alone, outside home or work, in front of the computer, late in the day and stress eating); and (iii) it is not known if the controls were weight stable [30]. 

Future work can investigate the association between dietary intake using omics techniques on HD symptomatology and outcomes. Additionally, future work, including further prospective studies, would be of value to ascertain whether dietary intake is associated with (delayed) disease onset and symptoms and improvement in quality of life. Furthermore, it would be interesting to investigate patients who have phenoconverted to observe any associations with dietary changes from their baseline CyFFQ. Future longitudinal investigations are needed to better understand the nutritional and anthropometric status changes that occur as the disease progresses. 

## 5. Conclusions

In summary, this study confirmed previous findings arguing that HD cases have a significantly higher energy intake than controls and revealed differences in macro and micronutrients and adherence to the MD, both between patients and controls and by HD symptom severity. These findings are important, as they provide evidence to further understand diet–disease associations and may guide future nutritional education efforts within this population group. Insights from research on diet in HD patients may pave the way for future, tailored dietary interventions, including the MD, which helps delay disease and symptom onset, decreases disease severity and improves the patients’ quality of life. 

## Figures and Tables

**Table 1:** Energy intake, macro and micronutrients of cases vs. controls.

	Cases (*n* = 36)		Controls (*n* = 37)		
	Median	IQR	Median	IQR	*p*-Value
Energy (kcal/day)	4592	3376	2488	1917	0.0002 *
Protein (% energy)	17.3	3.7	17.0	4.0	0.675
Fat (% energy)	41.8	7.8	42.4	10.9	0.651
CHO (% energy)	39.0	6.7	36.6	8.5	0.426
Starch (% energy)	13.5	6.6	12.2	4.6	0.284
Tot sug (% energy)	15.2	8.0	15.6	8.3	0.487
Sat fats (% energy)	13.5	3.6	13.4	3.2	0.903
MUFA (% energy)	15.0	4.1	15.5	5.7	0.089
PUFA (% energy)	6.4	2.8	6.4	1.7	0.304
Trans fats (% energy)	0.4	0.2	0.4	0.2	0.643
NSP (% energy)	1.1	0.6	1.6	0.8	0.034 *
Alcohol (% energy)	2.1	7.4	2.6	11.3	0.441
Cholesterol (mg/1000 kcal)	132.6	31.7	106.8	58.8	0.095
NSP (g/1000 kcal)	5.5	3.2	8.1	4.1	0.034 *
Fibre (g/1000 kcal)	6.9	4.1	9.9	5.5	0.028 *
Sodium (mg/1000 kcal)	990.9	269.3	915.9	344.0	0.142
Potassium (mg/1000 kcal)	1170.1	314.1	1377.8	292.0	0.053
Calcium (mg/1000 kcal)	372.1	134.4	402.6	159.4	0.274
Magnesium (mg/1000 kcal)	130.2	35.9	141.4	33.7	0.145
Phosphorus (mg/1000 kcal)	571.2	133.6	619.8	158.3	0.127
Iron (mg/1000 kcal)	5.2	1.6	5.7	2.8	0.185
Zinc (mg/1000 kcal)	4.2	1.2	4.0	1.1	0.956
Selenium (ug/1000 kcal)	18.8	7.1	16.8	6.4	0.125
Vit D (ug/1000 kcal)	0.7	0.2	0.7	0.5	0.947
Vit E (mg/1000 kcal)	3.6	2.1	3.4	3.1	0.956
Thiamin (mg/1000 kcal)	0.6	0.2	0.7	0.2	0.401
Riboflavin (mg/1000 kcal)	0.6	0.2	0.6	0.3	0.508
Vit B6 (mg/1000 kcal)	0.7	0.4	0.8	0.3	1.000
Vit B12 (ug/1000 kcal)	1.6	0.6	1.4	0.4	0.216
Trypto60 (mg/1000 kcal)	6.2	2.6	5.7	1.7	0.182
Folate (ug/1000 kcal)	64.7	29.8	79.5	33.0	0.077
Pantothenic acid (ug/1000 kcal)	2.1	0.5	2.0	0.5	0.920
Vit C (mg/1000 kcal)	27.1	30.1	34.5	36.4	0.145

EA: Energy Adjusted. CHO: Carbohydrates. Tot sug: Total Sugar. Sat fats: Saturated fats. MUFA: Monounsaturated fatty acids. PUFA: Polyunsaturated fatty acids. NSPs: Non-starch polysaccharides. Trypto60: Tryptophan. * Statistically significant *p*-values < 0.05.

**Table 2:** Energy intake, macro and micronutrients of asymptomatic HD patients and controls.

	Asymptomatic (*n* = 18)		Controls (*n* = 37)		
	Median	IQR	Median	IQR	*p*-Value
Energy (kcal/day)	3751	1894	2488	1917	0.028 *
Protein (% energy)	18.8	3.1	17.0	4.0	0.184
Fat (% energy)	42.8	6.4	42.4	10.9	0.628
CHO (% energy)	39.0	8.2	36.6	8.5	0.900
Starch (% energy)	12.5	7.0	12.2	4.6	0.698
Tot sug (% energy)	16.1	5.8	15.6	8.3	0.885
Sat fats (% energy)	14.5	3.8	13.4	3.2	0.419
MUFA (% energy)	15.7	2.9	15.5	5.7	0.590
PUFA (% energy)	7.5	2.6	6.4	1.7	0.032 *
Trans fats (% energy)	0.4	0.2	0.4	0.2	0.332
NSP (% energy)	1.3	0.6	1.6	0.8	0.156
Alcohol (% energy)	1.5	4.7	2.6	11.3	0.340
Cholesterol (mg/1000 kcal)	135.8	55.4	106.8	58.8	0.023 *
NSP (g/1000 kcal)	6.4	3.2	8.1	4.1	0.156
Fibre (g/1000 kcal)	8.0	4.3	9.9	5.5	0.161
Sodium (mg/1000 kcal)	1028.6	290.2	915.9	344.0	0.044 *
Potassium (mg/1000 kcal)	1219.2	450.2	1377.8	292.0	0.451
Calcium (mg/1000 kcal)	410.2	138.4	402.6	159.4	0.628
Magnesium (mg/1000 kcal)	132.8	32.3	141.4	33.7	0.928
Phosphorus (mg/1000 kcal)	615.8	141.9	619.8	158.3	0.578
Iron (mg/1000 kcal)	5.1	1.6	5.7	2.8	0.332
Zinc (mg/1000 kcal)	4.4	0.6	4.0	1.1	0.151
Manganese (mg/1000 kcal)	0.9	0.4	0.9	0.4	0.900
Selenium (ug/1000 kcal)	19.7	6.8	16.9	6.4	0.023 *
Vit D (ug/1000 kcal)	0.8	0.4	0.7	0.5	0.281
Vit E (mg/1000 kcal)	4.1	2.7	3.4	3.1	0.409
Thiamin (mg/1000 kcal)	0.7	0.3	0.7	0.2	0.928
Riboflavin (mg/1000 kcal)	0.7	0.2	0.6	0.3	0.389
Vit B6 (mg/1000 kcal)	0.7	0.3	0.8	0.3	0.857
Vit B12 (ug/1000 kcal)	1.7	0.3	1.4	0.4	0.052
Trypto60 (mg/1000 kcal)	6.8	2.1	5.7	1.7	0.007 *
Folate (ug/1000 kcal)	68.2	30.6	79.5	33.0	0.131
Pantothenic acid (ug/1000 kcal)	2.2	0.6	2.0	0.5	0.167
Vit C (mg/1000 kcal)	21.8	49.9	34.5	36.4	0.136

EA: Energy Adjusted. CHO: Carbohydrates. Tot sug: Total Sugar. Sat fats: Saturated fats. MUFA: Monounsaturated fatty acids. PUFA: Polyunsaturated fatty acids. NSP: Non-starch polysaccharides. Trypto60: Tryptophan. * Statistically significant *p*-values < 0.05.

**Table 3:** Energy intake, macro and micronutrients of symptomatic HD patients and controls.

	Symptomatic (*n* = 18)		Controls (*n* = 37)		
	Median	IQR	Median	IQR	*p*-Value
Energy (kcal/day)	5511	2907	2488	1917	0.0001 *
Protein (% energy)	16.5	4.2	17.0	4.0	0.518
Fat (% energy)	39.4	6.8	42.4	10.9	0.222
CHO (% energy)	38.8	5.5	36.6	8.5	0.243
Starch (% energy)	13.5	5.3	12.2	4.6	0.178
Tot sug (% energy)	14.6	8.5	15.6	8.3	0.323
Sat fats (% energy)	13.0	4.5	13.4	3.2	0.541
MUFA (% energy)	13.4	5.2	15.5	5.7	0.026 *
PUFA (% energy)	6.0	2.4	6.4	1.7	0.640
Trans fats (% energy)	0.4	0.2	0.4	0.2	0.829
Fibre (% energy)	1.3	0.7	1.9	1.1	0.031 *
Alcohol (% energy)	3.5	9.8	2.6	11.3	0.766
Cholesterol (g/1000 kcal)	125.6	35.6	106.9	58.8	0.653
NSP (g/1000 kcal)	5.5	2.9	8.1	4.1	0.042 *
Fibre (g/1000 kcal)	6.7	3.7	9.9	5.5	0.031 *
Sodium (mg/1000 kcal)	968.7	270.2	916.0	344.0	0.706
Potassium (mg/1000 kcal)	1155.7	185.3	1377.8	291.9	0.017 *
Calcium (mg/1000 kcal)	348.2	99.3	402.6	159.4	0.023 *
Magnesium (mg/1000 kcal)	115.8	38.1	141.7	33.7	0.022 *
Phosphorus (mg/1000 kcal)	503.8	84.2	619.8	158.4	0.002 *
Iron (mg/1000 kcal)	5.3	1.5	5.6	2.7	0.236
Zinc (mg/1000 kcal)	3.5	1.3	4.0	1.1	0.127
Manganese (mg/1000 kcal)	0.8	0.5	0.9	0.4	0.222
Vit D (ug/1000 kcal)	0.7	0.3	0.7	0.5	0.236
Vit E (mg/1000 kcal)	2.8	2.1	3.4	3.1	0.360
Thiamin (mg/1000 kcal)	0.6	0.2	0.7	0.2	0.146
Riboflavin (mg/1000 kcal)	0.5	0.1	0.6	0.3	0.052
Vit B6 (mg/1000 kcal)	0.7	0.5	0.8	0.3	0.857
Vit B12 (ug/1000 kcal)	1.3	0.8	1.4	0.4	0.942
Folate (ug/1000 kcal)	63.9	26.8	79.5	33.0	0.172
Pantothenic acid (ug/1000 kcal)	1.9	0.6	2.041	0.5	0.222
Vit C (mg/1000 kcal)	31.2	26.6	34.5	36.4	0.379

EA: Energy Adjusted. CHO: Carbohydrates. Totsug: Total Sugar. Satfats: Saturated fats. MUFA: Monounsaturated fatty acids. PUFA: Polyunsaturated fatty acids. NSP: Non-starch polysaccharides. * Statistically significant *p*-values < 0.05.

**Table 4:** Percent within recommendations of energy intake, macro and micronutrients and comparison between cases and controls.

		Cases (*n* = 36)			Controls (*n* = 37)			
	Recommendation *	% within Recommendation	Lower 95% CI	Upper 95% CI	% within Recommendation	Lower 95% CI	Upper 95% CI	*p*-Value (Fisher’s Exact Test)
Energy (kcal/day)	Males: 2500 kcal/day Females: 2000 kcal/day	8.3	2.6	23.6	29.7	16.9	46.7	0.035 *
Protein (% energy)	10–35% of E	97.2	81.7	99.6	97.3	82.2	99.6	1.000
Total fat (%energy)	20–35% of E	11.1	4.1	26.8	10.8	4.0	26.2	1.000
CHO (% energy)	45–65% of E	13.9	5.7	30.0	21.6	10.9	38.3	0.543
Sat Fats (% energy)	10% of E	5.6	1.3	20.5	8.1	2.5	23.0	1.000
MUFA (% energy)	__	__	__	__	__	__	__	__
PUFA (% energy)	6–11% of E	55.6	38.8	71.2	56.8	40.1	72.0	1.000
Trans Fats (% energy)	<1% of E	100.0	__	__	100.0	__	__	__
Cholesterol (mg/day)	≤300 mg/d	8.3	2.6	23.6	51.4	35.1	67.3	<0.0001 *
Fibre (g/day)	Males ≤50 years: ≥38 g/day (≥EAR) Males >50 years: ≥30 g/day (≥EAR) Females ≤50 years: ≥25 g/day (≥EAR) Females >50 years: ≥21 g/day (≥EAR)	66.7	49.4	80.4	59.5	42.7	74.3	0.630
Sodium (mg/day)	<2300 mg/day (≤UL)	8.3	2.6	23.6	45.9	30.3	62.4	<0.0001 *
Potassium (mg/day)	≥4700 mg/day (≥AI)	94.4	79.5	98.7	83.8	67.6	92.7	0.261
Calcium (mg/day)	Males ≤50 years: 1000–2500 mg/day (≥RDA) Males ≤50 years: 1000–2500 mg/day (≤UL) Males >50 years: 1000–2000 mg/day (≥RDA) Males >50 years: 1000–2000 mg/day (≤UL) Females ≤50 years: 1000–2500 mg/day (≥RDA) Females ≤50 years: 1000–2500 mg/day (≤UL) Females >50 years: 1200–2000 mg/day (≥RDA) Females >50 years: 1200–2000 mg/day (≤UL)	63.9	46.7	78.2	40.5	25.7	57.3	0.062
Magnesium (mg/day)	Males: ≥420 mg/day (≥EAR) Females: ≥420 mg/day (≥EAR)	80.6	63.8	90.7	56.8	40.1	72.0	0.043 *
Phosphorus (mg/day)	≥700 mg/day (≥EAR)	100.0	__	__	100.0	__	__	__
Iron (mg/day)	Males ≤50 years: 8–45 mg/day (≥RDA) Males ≤50 years: 8–45 mg/day (≤UL) Females ≤50 years: 18–45 mg/day (≥RDA) Females ≤50 years: 18–45 mg/day (≤UL) Females >50 years: 8–45 mg/day (≥RDA) Females >50 years: 8–45 mg/day (≤UL)	75.0	57.9	86.8	62.2	45.2	76.6	0.315
Zinc (mg/day)	Males: 11–40 mg/day (≥RDA) Males: 11–40 mg/day (≤UL) Females: 8–40 mg/day (≥RDA) Females: 8–40 mg/day (≤UL)	83.3	66.9	92.5	54.1	37.6	69.7	0.011 *
Vit D (ug/day)	15–100 ug/day (≥RDA) 15–100 ug/day (≤UL)	0.0	__	__	0.0	__	__	__
Vit E (mg/day)	15–100 mg/day (≥RDA) 15–100 mg/day (≤UL)	61.1	44.0	75.9	21.6	10.9	38.3	0.010 *
Thiamin (mg/day)	Males: ≥1.2 mg/day (≥EAR) Females: ≥1.1 mg/day (≥EAR)	94.4	79.5	98.7	89.2	73.8	96.0	0.674
Riboflavin (mg/day)	Males: ≥1.3 mg/day (≥EAR) Females: ≥1.1 mg/day (≥EAR)	94.4	79.5	98.7	81.1	64.7	90.9	0.152
Niacin (mg/day)	Males: 16–35 mg/day (≥EAR) Males: 16–35 mg/day (≤UL) Females: 14–35 mg/day (≥RDA) Females: 14–35 mg/day (≤UL)	47.2	31.2	63.8	56.8	40.1	72.0	0.486
Vit B6 (mg/day)	Males ≤50 years: 1.3–100 mg/day (≥RDA) Males ≤50 years: 1.3–100 mg/day (≤UL) Males >50 years: 1.7–100 mg/day (≥RDA) Males >50 years: 1.7–100 mg/day (≤UL) Females ≤50 years: 1.3–100 mg/day (≥RDA) Females ≤50 years: 1.3–100 mg/day (≤UL) Females >50 years: 1.5–100 mg/day (≥RDA) Females >50 years: 1.5–100 mg/day (≤UL)	94.4	79.5	98.7	75.7	58.9	87.1	0.046 *
Vit B12 (ug/day)	≥2.4 ug/day (≥EAR)	94.4	79.5	98.7	89.2	73.8	96.0	0.674
Folate (ug/day)	400–1000 ug/day (≥RDA) 400–1000 ug/day (≤UL)	33.3	19.6	50.6	10.8	4.0	26.2	0.025 *
Pantothenic acid (ug/day)	≥5 ug/day (≥AI)	86.1	70.0	94.3	51.4	35.1	67.3	0.002 *
Vit C (mg/day)	Males: 90–2000 mg/day (≥EAR) Males: 90–2000 mg/day (≤UL) Females: 75–2000 mg/day (≥RDA) Females: 75–2000 mg/day (≤UL)	69.4	52.2	82.6	59.5	42.7	74.3	0.465

CHO: Carbohydrates. Tot sug: Total Sugar. Sat fats: Saturated fats. MUFA: Monounsaturated fatty acids. PUFA: Polyunsaturated fatty acids. NSP: Non-starch polysaccharides. RDA: Recommended Dietary Allowances; the average daily dietary intake level sufficient to meet the nutrient requirements of nearly all (97–98%) healthy individuals in a group. It is calculated according to Estimated Average Requirement (EAR). EAR: Estimated Average Requirement. AI: Adequate Intake; Calculated if sufficient evidence is not available to establish an EAR, otherwise calculating an RDA. AI is believed to cover the needs of all healthy individuals in a group, but lack of data or uncertainty in the data prevents us from specifying with confidence the % of individuals covered by this intake. UL: Upper Intake Level; the highest level of daily nutrient intake that is likely to pose no risk of adverse health effects to almost all individuals in the general population. UL usually represents intake from food, water and supplements. Due to the lack of data, Uls could not be calculated for many of the micronutrients. In these cases, no upper intake limit was denoted in the definition of the Recommendation [19]. * Statistically significant *p*-values < 0.05.

**Table 5:** Adherence to the Mediterranean diet assessed using the MedDiet score.

MedDiet Score					
	Median	IQR	Mean	Std Dev.	Minimum and Maximum
Cases and Controls (*n* = 73)	32.1	7.1	31.5	5.4	12.0–42.0
Controls (*n* = 37)	31.1	8.1	31.2	4.5	23.0–40.0
Asymptomatic (*n* = 18) ^a^	31.1	6.1	29.3	6.2	12.0–39.0
Symptomatic (*n* = 18) ^b,c^	33.1	8.1	5.3	5.3	24.0–42.0

IQR: Interquantile range and Std Dev: Standard Deviation. a: *p*-value comparing asymptomatic cases to controls = 0.363; b: *p*-value comparing symptomatic cases to controls = 0.061; and c: *p*-value comparing symptomatic to asymptomatic cases = 0.024.

**Table 6:** Adherence to the Mediterranean diet assessed using MEDAS score.

MEDAS Score						
	Median	IQR	Mean	Std Dev.	Minimum and Maximum	*p*-Value
Cases and Controls (*n* = 73)	7.2	2.0	6.6	1.8	2.0–11.1	
Controls (*n* = 37)	8.2	2.0	7.2	1.8	3.0–11.1	
Asymptomatic (*n* = 18) ^a^	5.5	3.0	5.7	2.1	2.0–10.1	
Symptomatic (*n* = 18) ^b,c^	6.2	1.0	1.2	1.2	4.0–9.1	

IQR: Interquantile range and Std Dev: Standard Deviation. a: *p*-value comparing asymptomatic cases to controls = 0.014; b: *p*-value comparing symptomatic cases to controls = 0.066; and c: *p*-value comparing symptomatic to asymptomatic cases = 0.216.

## Data Availability

The data presented in this study are available on request from the corresponding author. The data are not publicly available due to privacy restrictions.

## Supplementary Materials

The following supporting information can be downloaded at: https://www.mdpi.com/article/10.3390/nu15051136/s1, Table S1: Demographic data of asymptomatic HD patients versus controls; Table S2: Demographic data of symptomatic HD patients versus controls; Table S3: Demographic data of asymptomatic HD patients versus symptomatic HD patients; Table S4: Criteria and scoring of the MedDiet score; Table S5: Criteria and scoring of the MEDAS score.
