# Supplementary material for: Investigating the Dietary Intake Using the CyFFQ Semi-Quantitative Food Frequency Questionnaire in Cypriot Huntington’s Disease Patients
Source: Nutrients. 2023 Feb 23;15(5):1136. doi: 10.3390/nu15051136 (PMC10005621; doi:10.3390/nu15051136)
Supplement: Supplementary file 1 [file nutrients-15-01136-s001.zip › nutrients-2197256-supplementary.pdf]

Table S1. Demographic and anthropometric data of asymptomatic HD patients versus controls

		Control (n=37)	Asymptomatic (n=18)	•p-value
ƚ **Age**	N	33	18	
	Median (IQR)	47 (23.2)	37 (11.7)	**0.026***
**Gender**				
Male	N (%)	14 (38%)	6 (33%)	1.000
Female	N (%)	23 (62%)	12 (67%)	
ƚ **Residence District**				
Nicosia	N (%)	19 (54%)	0 (0%)	
Limassol	N (%)	3 (9%)	3 (17%)	
Larnaca	N (%)	8 (23%)	9 (50%)	0.000
Ammochostos	N (%)	5 (14%)	4 (22%)	
Paphos	N (%)	0 (0%)	2 (11%)	
ƚ **Educational Level**				
Primary School	N (%)	1 (3%)	2 (11%)	
Lower Secondary	N (%)	5 (14%)	5 (28%)	
School		4 (11%)	6 (33%)	**0.014***
High school	N (%)	25 (71%)	5 (28%)	
Higher Education	N (%)			
ƚ **Marital Status**				
Married	N (%)	26 (70%)	9 (50%)	
Single	N (%)	8 (22%)	7 (38%)	0.150
Divorced	N (%)	0 (0%)	1 (6%)	
Widowed	N (%)	1 (3%)	1 (6%)	
ƚ **Weight**	N	32	18	
	Median (IQR)	76 (21)	68 (22)	**0.496**
ƚ **BMI**				
**Normal weight 20-24.9**	N (%)	9 (19%)	6 (13%)	
**Underweight ≤20**	N (%)	4 (8%)	2 (4%)	**0.977**
**Overweight 25-29.9**	N (%)	12 (25%)	6 (13%)	
**Obesity <30**	N (%)	9 (19%)	3 (6%)	
ƚ **Ever Smoked**				
No	N (%)	19 (54%)	8 (44%)	0.569
Yes	N (%)	16 (46%)	10 (56%)	
ƚ **Current Smoking**				
No	N (%)	23 (66%)	10 (56%)	
Yes	N (%)	6 (17%)	6 (33%)	0.444
Rarely	N (%)	2 (6%)	2 (11%)	

•Gender was compared for controls (n=37) and asymptomatic HD patients (n=18). Residence district, educational level, marital status, ever smoked and current smoking was compared for controls (n=35) and asymptomatic HD patients (n=18).

ƚ Indicates that there are missing values for the following demographics variables in the control group (n=35). *Indicates statistically significant results.

Table S2. Demographic data and anthropometric of symptomatic HD patients versus controls

		Control (n=37)	Symptomatic (n=18)	•p-value
**ł Age**	N	33	18	
	Median (IQR)	47 (23)	58 (17)	**0.041***
**Gender**				
Male	N (%)	14 (38%)	7 (39%)	1.000
Female	N (%)	23 (62%)	11 (61%)	
**ł Residence District**				
Nicosia	N (%)	19 (54%)	7(39%)	
Limassol	N (%)	3 (9%)	4 (22%)	
Larnaca	N (%)	8 (23%)	4(22%)	0.488
Ammochostos	N (%)	5(14%)	3 (17%)	
Paphos	N (%)	0 (0%)	0 (0%)	
**ł Educational Level**				
Primary School		1 (3%)	7 (39%)	
Lower Secondary	N (%)	5 (14%)	3 (17%)	
School	N (%)	4 (11%)	4 (22%)	0.000
High school		25 (71%)	4 (22%)	
Higher Education	N (%)			
	N (%)			
**ł Marital Status**				
Married	N (%)	26 (70%)	9 (50%)	
Single	N (%)	8 (22%)	4 (22%)	**0.035***
Divorced	N (%)	0 (0%)	3 (17%)	
Widowed	N (%)	1 (3%)	2 (11%)	
**ł Weight**	N	33	18	**0.030***
	Median (IQR)	76 (21)	64 (18)	
**ł BMI**				
**Normal weight 20-24.9**	N (%)	9 (19%)	6 (13%)	
**Underweight ≤20**	N (%)	4 (8%)	2 (4%)	0.977
**Overweight 25-39.9**	N (%)	12 (25%)	6 (13%)	
**Obesity >30**	N (%)	6 (13%)	3 (6%)	
**ł Ever Smoked**				
No	N (%)	19 (54%)	7 (39%)	0.555
Yes	N (%)	16 (46%)	10 (56%)	
**ł Current Smoking**				
No	N (%)	23 (66%)	11 (61%)	
Yes	N (%)	6 (17%)	5 (28%)	0.764
Rarely	N (%)	2 (6%)	1 (6%)	

•Gender was compared for controls (n=37) and asymptomatic HD patients (n=18). Residence district, educational level, marital status, ever smoked and current smoking was compared for controls (n=35) and asymptomatic HD patients (n=18).

ł Indicates that there are missing values for the following demographics variables in the control group (n=35). *Indicates statistically significant results.

Table S3. Demographic data and anthropometric of Asymptomatic versus Symptomatic HD patients.

		Asymptomatic (n=18)	Symptomatic (n=18)	•p-value
**Age**	N	18	18	
	Median (IQR)	35 (12)	58 (17)	**<0.00001***
**Gender**				
Male	N (%)	6 (33%)	7 (39%)	1.000
Female	N (%)	12 (67%)	11 (61%)	
**Residence District**				
Nicosia	N (%)	0 (0%)	7 (39%)	
Limassol	N (%)	3 (17%)	4 (22%)	
Larnaca	N (%)	9 (50%)	45 (22%)	**0.018***
Ammochostos	N (%)	4 (22%)	3 (17%)	
Paphos	N (%)	2 (11%)	0 (%)	
**Educational Level**				
Primary School	N (%)	2 (11%)	7 (39%)	
Lower Secondary	N (%)	5 (28%)	3 (17%)	
School		6 (33%)	4 (22%)	0.327
High school	N (%)	5 (28%)	4 (22%)	
Higher Education	N (%)			
**Marital Status**				
Married		9 (50%)	9 (50%)	
Single		7 (39%)	4 (22%)	0.607
Divorced		1 (6%)	3 (17%)	
Widowed		1 (6%)	2 (11%)	
[1] **Weight**	N	18	15	
	Median (IQR)	68 (22)	64 (18)	0.289
**BMI**				
**Normal weight 20-24.9**	N (%)	6 (21%)	2 (7%)	
**Underweight ≤20**	N (%)	2 (7%)	5 (17%)	0.267
**Overweight 25-29.9**	N (%)	6 (21%)	4 (14%)	
**Obesity >30**	N (%)	3 (10%)	1 (3%)	
**Inheritance**				
Paternal	N (%)	6 (33%)	4 (22%)	
Maternal	N (%)	10 (56%)	7 (39%)	0.181
Unknown	N (%)	2 (11%)	7 (39%)	
**Ever Smoked**				
No	N (%)	8 (44%)	7 (39%)	1.000
Yes	N (%)	10 (56%)	10 (56%)	
**Current Smoking**				
No	N (%)	10 (56%)	11 (61%)	
Yes	N (%)	6 (33%)	5 (18%)	1.000
Rarely	N (%)	2 (11%)	1 (18%)	

Gender, residence district, educational level, marital status, inheritance, ever smoked and current smoking was compared for asymptomatic HD patients (n=18) and symptomatic HD (n=18) patients.

*Indicates statistically significant results.

Indicates that there are missing values for the following demographics variables in the case group (n=15).

Table S4. Criteria and scoring of the MedDiet score

Food Indexes	Frequency of consumption (servings/week or otherwise stated)					
Non-refined cereals (whole grain bread, pasta, rice etc.)	Never	1-6	7-12	13-18	19-31	>32
**Non-refined cereals score**	0	1	2	3	4	5
Potatoes	Never	1-4	5-8	9-12	13-18	>18
**Potatoes score**	0	1	2	3	4	5
Fruits	Never	1-4	5-8	9-15	16-21	>22
**Fruits score**	0	1	2	3	4	5
Vegetables	Never	1-6	7-12	13-20	21-32	>33
**Vegetables score**	0	1	2	3	4	5
Legumes	Never	<1	1-2	3-4	5-6	>6
**Legumes score**	0	1	2	3	4	5
Fish	Never	<1	1-2	3-4	5-6	>6
**Fish score**	0	1	2	3	4	5
Red meat and products	0	1	2	3	4	5
**Red meat and products score**	≤1	2-3	4-5	6-7	8-10	>10
Poultry	5	4	3	2	1	0
**Poultry score**	≤3	4-5	5-6	7-8	9-10	>10
Full fat dairy products (cheese, yoghurt, milk)	≤10	11-15	16-20	21-28	29-30	>30
**Full fat diary product score**	5	4	3	2	1	0
Olive oil in cooking (times/week)	Never	Rare	<1	1-3	3-5	Daily
**Olive oil in cooking score**	0	1	2	3	4	5
Alcoholic beverages (ml/day, 100 ml=12g ethanol)	<300<br>5	300<br>4	400<br>3	500<br>2	600<br>1	>700 or 0<br>0
**Alcoholic beverages score**	5	4	3	2	1	0

Table S5. Criteria and scoring of the MEDAS score

Questions	Criteria for 1 point
1. Do you olive oil as main culinary fat?	Yes
2. How much olive oil do you consume in a given day (including oil used for frying, salads, out-of-house meals etc.)?	≥4 tbsp
3. How many vegetables serving do you consume per day?	≥2
4. How many fruit units (including natural fruit juices do you consume per day?	≥3
5. How many servings of red meat, hamburger or meat products (ham, sausage etc.) do you consume per day?	<1
6. How many servings of butter, margarine or cream do you consume per day?	<1
7. How many sweets or carbonated beverages do you drink per day?	<1
8. How much wine do you drink per week?	≥7 glasses
9. How many servings of legumes do you consume per week?	≥3
10. How many servings of fish or shellfish do you consume per week?	≥3
11. How many times per week do you consume commercial sweets or pastries (not homemade), such as cakes, cookies, biscuits or custard?	<3
12. How many servings of nuts (including peanuts) do you consume per week?	≥3
13. Do you preferentially consume chicken, turkey or rabbit meat instead of veal, pork, hamburger or sausage?	Yes
14. How many times per week do you consume vegetables, pasta, rice or other dishes seasoned with sofrito (sauce made with tomato and onion, leek or garlic and simmered with olive oil)?	≥2
